# Introgression of rye chromosome arm 1RS enhances climate resilience in German winter wheat

**DOI:** 10.1007/s00122-026-05314-6

**Published:** 2026-07-28

**Authors:** Yeneneh Bekele-Reba, Lorenz Bülow, Anne Zaar, Michael Koch, Adalbert Bund, Carsten Reinbrecht, Jost Dörnte, Hubert Kempf, Josef Holzapfel, Marco Stucke, Jörg Plieske, Lorenz Hartl, Volker Mohler, Stefan Streng, Benjamin Stich, Bernd Hackauf

**Affiliations:** 1 Julius Kühn Institute – Federal Research Center on Cultivated Plants, Institute for Breeding Research on Agricultural Crops, Rudolf-Schick-Platz 3a, 18190 Sanitz, Germany; 2 Professorship Utilization of Plant Genetic Resources for Breeding Purposes, University of Rostock, Justus‑Von‑Liebig‑Weg 6, 18059 Rostock, Germany; 3 Deutsche Saatveredelung AG, Weissenburger Straße 5, 59557 Lippstadt, Germany; 4 Institute for Crop Science and Plant Breeding, Bavarian State Research Center for Agriculture, Am Gereuth 8, 85354 Freising, Germany; 5 Saatzucht Streng-Engelen GmbH & Co. KG, Aspachhof, 97215 Uffenheim, Germany; 6 SECOBRA Saatzucht GmbH, Feldkirchen 3, 85368 Moosburg, Germany; 7 TraitGenetics Section, SGS INSTITUT FRESENIUS GmbH, Am Schwabeplan 1B, Gatersleben, 06466 Seeland, OT, Germany

## Abstract

**Key message:**

We provide quantitative evidence that rye chromosome arm 1RS exerts background- and context-dependent effects on yield, stability, and grain protein content in contemporary elite winter wheat. Integrated multi-environment analyses demonstrate that translocation lines exhibit pronounced yield stability across 14 contrasting environments, with ‘Insave’- and ‘Petkus’-derived segments contributing through distinct performance profiles relevant for climate-resilient wheat breeding.

**Abstract:**

Climate extremes increasingly threaten wheat production and yield stability globally. Rye (*Secale cereale* L.) chromosome arm 1RS has long been deployed in wheat breeding, yet its agronomic performance under drought and its interaction with elite genetic backgrounds remain insufficiently characterized. We evaluated 1RS translocations in elite German winter wheat using integrated molecular diversity analyses, multi-environment field phenotyping, and genomic modeling. Under near-optimal precipitation in 2021, matching the 1961–1990 reference period, the investigated winter wheat panel exhibited considerable genetic variation for yield and agronomic traits, indicating that high yield potential is still present in current German winter wheat breeding germplasm. In contrast, severe drought conditions in 2022 resulted in a 13.7% average yield decline, underscoring the sensitivity of current germplasm to drought stress. We show that 1RS translocations exhibit background-dependent effects on grain yield and stability. In particular, T1AL.1RS rye translocation originating from ‘Insave’ rye and stacked T1AL.1RS/T1BL.1RS translocations combining ‘Insave’ and ‘Petkus’ rye segments demonstrated favorable yield performance under drought conditions, although effects were context-dependent. Genetic modeling confirmed a significant interaction between 1RS translocations and the wheat genetic background, indicating that deployment of 1RS requires consideration of recipient genetic background. Our results highlight substantial response diversity within elite germplasm and demonstrate that targeted introgression of rye chromatin can contribute to improved climate resilience when systematically introgressed into adapted genetic backgrounds. The robust multi-environment field evaluation provides a strong foundation for interpreting translocation effects in modern elite germplasm. Complementary trait-level analyses, particularly root phenotyping and evaluation of double 1RS translocations within uniform genetic backgrounds, will further elucidate the physiological mechanisms underlying the observed responses.

**Supplementary Information:**

The online version contains supplementary material available at 10.1007/s00122-026-05314-6.

## Introduction

Climate change is a critical factor adversely affecting global food security through higher average temperatures and more frequent, severe droughts (Kumar et al. 2020). Among staple crops, common wheat (*Triticum aestivum* L.) is pivotal due to its global relevance and nutritional importance (Tadesse et al. 2019; Langridge and Reynolds 2021). Projections suggest that climate change could reduce global wheat yields by up to 40% toward the end of this century, posing a serious threat to food security in the world’s major wheat-producing countries even when adapted agricultural practices are applied (Hultgren et al. 2025). Europe is one of the main wheat-growing regions globally and has experienced greater and more pervasive climate variability over the last decade compared with the long-term average (Pasqui and Di Giuseppe 2019). In a changing climate, water deficit, alongside heat stress, is a leading constraint on crop growth and yield (Langridge et al. 2021). This was evident in 2022, when 36% of Europe experienced below-average precipitation, causing soil-moisture deficits; in the same year, about 17% of the European Union’s (EU) vegetation and crops showed adverse drought effects (https://climate.copernicus.eu/esotc/2022/drought).

Germany is the second-largest wheat producer in the EU, after France, contributing 3.4% to global wheat output between 2000 and 2023. During this period, German production reached 556.4 million tons (FAOSTAT 2025), a substantial share given the country’s moderate size and temperate climate. Analysis of long-term value for cultivation and use (VCU) trials conducted by the German Federal Plant Variety Office have demonstrated modest but consistent genetic gains in winter wheat yield from 1983 to 2016, alongside a slight increase in year-to-year yield variance (Hadasch et al. 2020). Importantly, water deficits during key growth stages were identified as significant drivers affecting both mean yield and its stability. This vulnerability persists even in currently advantaged regions, as targeted adaptation to emerging climate-related risks has only recently been prioritized in breeding programs (Hultgren et al. 2025). Consequently, the development of resilient cultivars is a key priority for wheat production in the EU and genotype-by-environment (G × E) interactions, already a cornerstone of plant breeding, are of even greater importance under ongoing climate change. Genotype–environment interactions often cause changes in the ranking of candidate lines when different drought-related indices or environments are used, thereby complicating the identification of truly drought-tolerant genotypes (Semahegn et al. 2020).

Recently, concerns on a decrease in response diversity among European wheat cultivars have been raised and suggested to be the reason for the reduced yield stability in European wheat trials (Kahiluoto et al. 2019). Indeed, a genome-wide association study previously identified two haplotypes on chromosome 5B that have been selected in European winter wheat varieties to improve flowering time, which lead to an undesirable reduction in root dry mass (Voss-Fels et al. 2017). Deep and persistent root systems are critical for maintaining water uptake under drought (Langridge and Reynolds 2021; Bapela et al. 2022). Optimizing the uptake of water and nutrients by an efficient root system is therefore a breeding goal with major importance to (i.) improve drought stress tolerance in European winter wheat and (ii.) secure the wheat productivity in Europe.

Rye (*Secale cereale* L.) is closely related to wheat and exhibits a particularly efficient root system (Dittmer 1937; Paponov et al. 1999), recovering up to 93% of available nitrate in field conditions compared with 57% for wheat (Yeo et al. 2014). Given that crosses between rye and wheat are feasible (Thompson 1926), rye offers transferable alleles and chromosomal segments to enhance wheat root performance. Wheats carrying translocations from rye, especially those with the short arm of chromosome 1 (1RS), have been integrated into wheat breeding programs globally mainly due to their contribution to resistance to biotic stress factors and agronomic performance (Crespo-Herrera et al. 2017). Beyond their well-documented role as carriers of resistance genes against a wide range of pathogens and pests, including stem rust, powdery mildew, greenbug, and wheat curl mite, 1RS translocations have increasingly been discussed in the context of abiotic stress adaptation. However, their contribution to drought resilience, yield stability, and genotype-by-environment interactions remains less systematically explored, particularly in modern breeding germplasm. Rye translocations, in particular, provide a means to compensate for the reduced root biomass observed in elite wheat germplasm. Indeed, studies on a series of near isogenic lines with the 1RS translocation from ‘Petkus’ rye in the genetic background of the spring wheat variety ‘Pavon76’ have shown that the T1BL.1RS translocation in ‘Pavon76’ leads to a more extensive root system and higher root biomass (Ehdaie et al. 2003; Sharma et al. 2010). Recently, evidence has been reported that dosage differences in a cluster of monocot-specific 12-OXOPHYTODIENOATE REDUCTASE genes from subfamily III (OPRIII) modulate key differences in root architecture of this translocation wheat (Gabay et al. 2023). Another translocation, T1AL.1RS, originates from Argentinian ‘Insave’ rye, is globally less frequently used compared to T1BL.1RS (Schlegel 2023), and has been only recently introduced in German wheat breeding. Although the 1RS translocations are on a global level widely used in wheat breeding (Han et al. 2025), their agronomic effects are highly context-dependent and not yet fully understood. A publicly available but unpublished dataset from German Federal Variety Trials during the drought year 2018 is referenced as supplementary information by Rabanus-Wallace et al. (2021). Briefly, this series of trials identified the bread wheat cultivar ‘Asory,’ which carries the T1AL.1RS translocation, outperforming all other tested bread wheat varieties across 26 German locations during the extreme drought of 2018. In contrast, the milling wheat cultivar ‘Kamerad,’ which carries the genetically distinct T1BL.1RS translocation, failed to reach comparable yield levels. These contrasting performances suggest that genetic differences between 1RS variants, alongside background effects, may influence drought resilience in German winter wheat. Several studies reported yield advantages and improved stress adaptation associated with 1RS introgressions, often linked to enhanced root development and water-use efficiency (Moreno-Sevilla et al. 1995; Ehdaie et al. 2003; Hoffmann 2008; Howell et al. 2014). However, other studies reported that the agronomic value of the 1RS translocation is not universally consistent. In some genetic backgrounds and environments, neutral or even negative yield effects have been reported, indicating strong genotype-by-environment interactions (Mathews et al. 2008; Pinto et al. 2010; Peake et al. 2011). Consistent with this view, Lelley et al. (2004) suggested that environmental stability may depend more strongly on the wheat genetic background than on the presence of the translocation itself. Despite these observations, the interaction between 1RS introgressions and the polygenic wheat background has rarely been quantified in modern elite winter wheat germplasm evaluated across multiple environments, and systematic comparisons of different 1RS translocations in the context of a detailed assessment of genotype-by-environment interactions (GEIs) remain limited. To address this gap, we conducted a comprehensive molecular and phenotypic analysis of a panel of modern German winter wheat to evaluate the agronomic relevance of both major 1RS translocations under variable environmental conditions. Our objectives were to (1) assess the frequency and nature of wheat–rye translocations in German winter wheat breeding programs, (2) evaluate stability and adaptability of winter wheat genotypes carrying wheat–rye translocations, and (3) investigate the extent to which the modern winter wheat elite lines allow for selection for climate resilience.

## Materials and methods

### Plant materials

The plant material consisted of *n* = 509 elite German winter wheat inbreds from three commercial and one public winter wheat breeding program (Supplementary Table 1). The material had been advanced within the breeding programs according to agronomic and quality criteria, and entries were then selected by the participating programs to represent relevant pedigrees, recent breeding cycles, and crosses in which putative rye translocations were expected based on pedigree information. The presence of rye chromatin was evaluated within the present study. Accordingly, the panel reflects the current German winter wheat breeding germplasm. Three released bread wheat varieties ‘Asory’ and ‘RGT Reform’ (quality class A, bread wheat), and ‘Kamerad’ (quality class B, milling wheat) were used as checks. Further 27 released varieties with E, A, B, and C baking quality grades of the German wheat classification system (Laidig et al. 2022) were also included. The A. E. Watkins landrace core collection of 119 bread wheat (Wingen et al. 2014, 2017) was included in our analysis as reference for genetic diversity studies. Elite breeding lines described in this study are proprietary to Deutsche Saatveredelung AG, Bavarian State Research Center for Agriculture, Saatzucht Streng-Engelen GmbH & Co. KG, and SECOBRA Saatzucht GmbH.

### Phenotyping

The field experiments were conducted at seven locations in Germany in the 2021 and 2022 cropping seasons. The field locations were Ranzin, (RAN, 53.9°N, 13.5°E), Aspachhof, (ASP, 49.6°N, 10.1°E), Groß Lüsewitz (GLW, 54.0°N, 12.3°E), Leutewitz (LEU, 51.0°N, 13.6°E), Weddegast (WED, 51.7°N, 11.8°E), Asendorf (ASD, 52.7°N, 9.0°E), and Feldkirchen (FDK, 48.1°N, 11.7°E). The location × year combinations are subsequently referred to as ‘environments.’ With an average of 805 l/m^2^, annual precipitation in 2021 was close to the average for both reference periods 1961–1990 (789 l/m^2^) and 1991–2020 (791 l/m^2^) in Germany. In 2022, average precipitation in Germany was with 670 l/m^2^ about 15% less compared to the reference period 1961–1990 (Güntner et al. 2023). A total of 320 entries, including repeated checks, were tested at each test environment. The genotype overlap between the two years was 101 genotypes. Entries were allocated to field trials that were set up as α-lattice designs with two replicates per environment, on 6 and 11 m^2^ plots, connected by released cultivars as checks and including incomplete blocks to account for spatial field variation. Field trials were managed according to integrated plant protection principles, including standard herbicide, fungicide, and fertilizer applications appropriate for the respective site and year. Phenotypic data were collected on four agronomic and quality traits (grain yield (GYD, Mg ha^−1^), heading date (HDT, days from sowing), plant height (PHT, cm), and grain protein content (GPC, % protein weight per seed dry weight). Grain protein content (GPC) was determined by near-infrared reflectance (NIR) spectroscopy using a whole-grain calibration developed within the BRIWECS project consortium. Reference values were obtained by wet-chemical analysis following ICC Standard 105/2. Spectral preprocessing and model development were performed in OPUS QUANT2 (Bruker Optics). The calibration showed high predictive accuracy across the observed GPC range (6.08–19.72%), with a coefficient of determination (R^2^) of 0.98 and a root-mean-square error of estimation (RMSEE) of 0.31. Cross-validation yielded R^2^cv = 0.98 and a root-mean-square error of cross-validation (RMSECV) of 0.33, corresponding to residual prediction deviation (RPD) values > 7, which indicates excellent predictive performance. Calibration and cross-validation plots are provided in Supplementary Fig. 1. The yield-protein derived index grain protein deviation (PDV), yield deviation (YDV), equal weight PDV and YDV (EWD), and equal weight GPC and GYD (EPY) were computed according to Rapp et al. (2018). PDV quantifies the deviation of a genotype’s actual grain protein concentration from the value expected based on its grain yield. It therefore describes the ability of a genotype to accumulate protein independently of yield level.

### Genotypic data and population structure

The plant materials were genotyped using the 25 k Illumina iSelect SNP array by SGS TraitGenetics (Gatersleben, Germany). SNP markers were selected from the 90 K Infinium array, two proprietary TraitGenetics wheat arrays (135 K Axiom and 12 K Infinium), and the 35 K Axiom wheat breeder array (Shahinnia et al. 2022). The final data set was filtered for maximum number of two alleles, 20% maximum missing data per marker, minimum gene diversity of 5%, and 20% maximum missing data per genotype using R package SelectionTools version 21.3 (Hofheinz and Frisch 2014). Finally, from 20,954 SNPs and 509 genotypes, a total number of 505 genotypes and 17,301 quality filtered polymorphic SNPs with known positions in the reference sequence v1.0 of the Chinese Spring genome assembly (IWGSC 2018) were available for population structure analysis through cluster and principal coordinate analysis (PCoA) based on the modified Rogers’ distances between the individuals (Rogers 1972). In contrast to principal component analysis (PCA), which is based on variance–covariance structures of marker data, principal coordinate analysis (PCoA) was performed on genetic distance matrices (modified Rogers’ distance), allowing a direct representation of pairwise genetic relationships among genotypes.

For genetic diversity analyses of the elite winter wheats including the A. E. Watkins landrace core collection as a reference, we calculated the genetic distance matrix using the provesti.dist() function (Prevosti et al. 1975) from the *poppr* R package version 2.9.6 (Kamvar et al. 2014). Genotypes carrying rye translocations were identified using (i.) grain storage protein loci (Kozub et al. 2018), (ii.) the *SCM9* microsatellite marker (Weng et al. 2007), (iii.) a SNP marker for T1BL.1RS (Zhao et al. 2019) as well as (iv.) presence-absence variation of SNP markers specific for the short arms of wheat chromosomes 1A and 1B. The R package *snpReady* version 0.9.7 (Granato et al. 2018) was employed to calculate means of the observed heterozygosity (H_o_), the expected heterozygosity (H_e_), the minor allele frequency (MAF), the polymorphism information content (PIC), and the inbreeding coefficient F. Gene diversity (expected heterozygosity, He) was calculated as a measure of allele frequency-based diversity across loci and used as a filtering criterion to remove markers with low polymorphism. It is important to distinguish between expected heterozygosity (He), which reflects allele frequency-based diversity at the population level, and observed heterozygosity (Ho) at the individual level. Because the analyzed material consists predominantly of advanced breeding lines (generations F5–F6), a certain level of residual heterozygosity is expected and does not contradict the classification as inbred material. Because diversity metrics depend on sample size, 50 random subsets (*n* = 119 each) were drawn from the elite panel to match the size of the Watkins collection, and mean diversity estimates were computed across subsamples. A principal component analysis was performed after mean imputation. In contrast to principal component analysis (PCA), which is based on variance–covariance structures of marker data, principal coordinate analysis (PCoA) was performed on genetic distance matrices (modified Rogers’ distance), allowing a direct representation of pairwise genetic relationships among genotypes. The analysis of molecular variance (AMOVA) (Excoffier et al. 1992) was conducted with a priori grouping of the genotypes according to their origin using the R package *poppr* (Kamvar et al. 2014).

### Phenotypic data analysis

The best linear unbiased estimates (BLUEs) for each genotype for the traits were obtained based on restricted maximum likelihood (REML) assuming fixed genotype effects, while the other variance sources were considered as random effects using the following equation (Eq. 1):

1 $$y = \mu + g + e + g:e + e:r + b:e:r + \varepsilon$$

where y is the phenotypic observation, *µ* the overall mean, g the effect of the genotype, e the effect of the environment, g:e is the genotype-by-environment interaction, e:r the replication nested within environment effect, b:e:r the block effect nested within replication and environment, and ε the residual. Estimates for variance components were computed assuming a random model in the above classical one stage equation (Eq. 1). The BLUEs from Eq. 1 were used for downstream analyses.

The broad-sense heritability (*H*^2^) describes the proportion of total phenotypic variance attributable to genetic variance and should therefore be interpreted in relation to the overall phenotypic variance observed across environments. *H*^2^ was computed using Eq. 2 (Piepho and Möhring 2007):

2 $$H^{2} = \left[ {\frac{{\sigma_{g}^{2} }}{{\sigma_{g}^{2} + \sigma_{m}^{2} }}} \right]$$

where ${\sigma}_{m}^{2}$ is the square of the average standard error of the adjusted treatment means as the masking variance and ${\sigma g}^{2}$ is the genetic variance. All phenotypic data analysis and cleaning were performed using the statistical software R (R Core Team 2021), the package asreml-r 4.1.0.176, and the sommer package version 4.3.2 (Butler et al. 2023). We used the additive main effects and multiplicative interaction (AMMI) model to partition genotype, environment, and genotype × environment (G × E) effects and employed performance stability analysis to the GYD data of 101 genotypes, which were tested consistently across the 14 environments. The stability variance for genotypic groups was calculated as described in Mühleisen et al. (2014), which can be interpreted analogously to the stability variance described by Shukla (1972). The best linear unbiased predictions (BLUPs) were obtained using the general mixed model (Eq. 3; Piepho 1994):

3 $$y = \mu + g + e + r + \varepsilon$$

where y, *µ, g, e,* and ε are as described in Eq. 1. Except for the fixed effect of the environment, the rest effects were considered random. Eleven parametric and 9 nonparametric stability measures (Baraki et al*.* 2024, Supplementary Table 5) were computed and plotted using R. The parametric set comprised ASV (AMMI stability value), EV (sums of the averages of the squared eigenvector values), SIPC (sums of the absolute values of the IPC scores), ZA (absolute value of the relative contribution of IPCs to the interaction), CV/CVi (coefficient of variation), Shukla’s variance (σ^2^), Wricke’s ecovalence (Ecoval), the regression coefficient (*bᵢ*), the deviation from regression (*Sᵢⱼ*), WAASB (weighted average of absolute scores), and HMRPGV (harmonic mean of the relative performance of genotypic values). The nonparametric set included the rank-based statistics S1, S2, S3, and S6, Thennarasu’s statistics N1–N4, and Kang’s rank sum (KR). WAASB (Olivoto and Lúcio 2020), derived from a mixed-model framework integrating AMMI and BLUP features, was emphasized as the primary stability statistic because it captures multi-dimensional GEI effects more comprehensively than classical AMMI approaches based solely on the first Interaction Principal Component Axis. To simultaneously select for yield and stability, the WAASBY superiority index was calculated by integrating mean yield and WAASB scores using predefined weights. Spearman’s rank correlations and hierarchical cluster analysis were performed to assess concordance among stability indices and to group genotypes with similar performance–stability profiles.

### Evaluating background-dependent effects of the 1RS translocation through genomic prediction

For this analysis, we used the following genomic prediction models:

4 $$y = \mu + t + g + \varepsilon$$

where *y* is the grain yield of the genotype. *μ* is the overall mean, *t* is the fixed effect of the translocation, *g* is the random effect of genotype based on the genomic relationship matrix K, and *ε* is the residual error.

5 $$y = \mu + t + g + \left( {t \times g} \right) + \varepsilon$$

where (*t* × *g*) is the random interaction effect between translocation and genotype background, modeled with the interaction kernel *K*_*tg*_, and *ε* is the residual error.

Both models were compared based on a likelihood ratio test.

## Results

### Rich genetic diversity in modern winter wheat

In a first step, we compared the genetic diversity and structure of the 505 current German winter wheat breeding lines and released cultivars with that of 119 inbreds of the A.E. Watkins landrace core collection based on SNP genotyping data. The appropriateness of the chosen SNP marker array for genetic analyses was confirmed by the similar PIC and MAF values of the genotype sets.

**Fig. 1:**
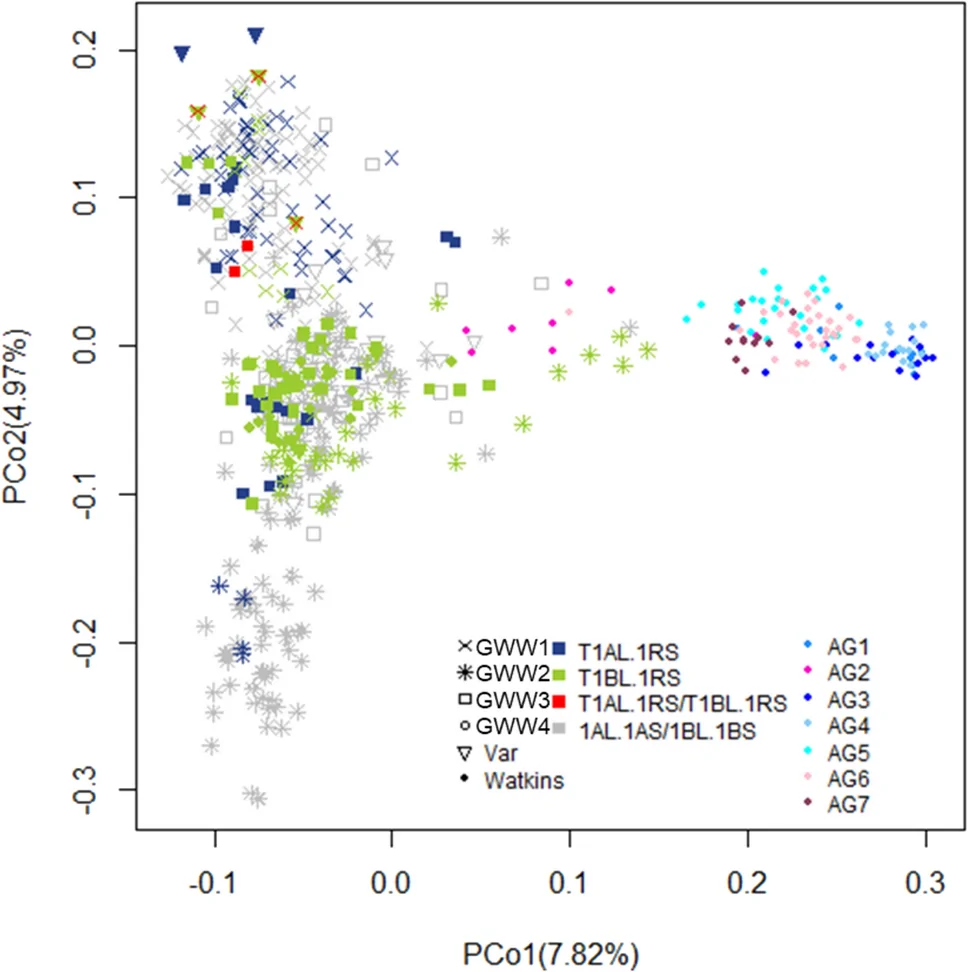
Principal coordinate analysis of 505 elite winter wheat genotypes and 119 inbreds of the Watkins collection of different ancestral groups (AG1-7) based on modified Roger’s distance calculated from 17,301 SNP marker loci. PCo1 and PCo2 are the first and second principal coordinate, respectively. The numbers in parentheses refer to the proportion of variance the principal coordinates explain. Color codes indicate genetic groups, while the shapes indicate the German winter wheat breeding programs (GWW1-4): and Var, released varieties and Watkins, Watkins collection. Color codes indicate genetic groups. T1AL.1RS (1RS is translocated on the long arm of wheat chromosome 1A designated as 1AL), T1BL.1RS (1RS is translocated on the long arm of wheat chromosome 1B designated as 1BL), T1AL.1RS/T1BL.1RS (genotypes with both translocations: T1AL.1RS and T1BL.1RS), and 1AL.1AS/1BL.1BS (without the 1RS translocations). 1RS, the short arm of rye chromosome 1R

Of the 505 winter wheat entries, 14.6% and 21.7% carried T1AL.1RS and T1BL.1RS, respectively (Supplementary Table 1). In 1% of the cases, both rye translocation segments were detected. The first two axes of a principal coordinate analysis (PCoA) including the core set of the A.E. Watkins landraces core collection and the modern winter wheat set accounted for 12.8% of the genetic differences in the investigated germplasm (Fig. 1). PCo1 and PCo2 separated two of the German winter wheat breeding programs, while genotypes of three programs clustered together. The frequency of rye translocations was not uniformly distributed among the studied breeding programs; two programs covered 29.5% of rye translocations, while the other two programs accounted for 66.1% of all translocations. The German winter wheats were distinct from the landraces, except for a few entries from ancestral group 2 (AG2, South Europe, Asia). Noteworthy, the variance of the A.E. Watkins landrace core collection was largely captured by PCo1, while the German winter wheats were separated by PCo2 which explained almost 5% of the variance. For the analyses of molecular variance (AMOVA), we referred to the whole set of the German winter wheat panel and the core set of the Watkins landraces collection as two genetic groups. The breeding lines from four different German winter wheat breeding programs (GWW1-4) and the released varieties in comparison with the landraces were considered as different populations within the two genetic groups (Table 2). According to the AMOVA, most of the molecular variation (69.50%) was found among individuals, while variation among the six populations within the two germplasm groups explained 6.2% of the total genetic diversity (Table 1). The molecular variance between German winter wheat and the landrace group was not significantly different and explained 15.7% of the total genetic diversity. The same trend was also indicated by the minor allele frequency (MAF) and expected heterozygosity (H_e_). Furthermore, the observed heterozygosity (H_o_) for all germplasm groups was low and the inbreeding coefficient (*F*_*IS*_) was high, as would be expected for a self-pollinating species, although these values differed for each genotype group. The observed levels of heterozygosity are consistent with the use of advanced breeding material rather than fully homozygous cultivars, reflecting residual segregation in early inbred generations and the deliberate inclusion of diverse breeding lines. The released German winter wheat cultivars and the landraces had the lowest H_o_ values and the highest *F*_*IS*_ values (Table 2). The resampled elite subsets showed a similar magnitude of diversity (PIC = 0.26, He = 0.37) to the Watkins collection (PIC = 0.29, He = 0.37)(Table 2), indicating that modern breeding has not resulted in a pronounced reduction of diversity at the assayed loci.

**Table 1:** Analysis of molecular variance in modern German winter wheat and the A.E. Watkins landrace core collection

Source of variation	*d.f*	Sum of square	Variance component sigma	Percent variation	*P*
Among groups	1	623,107.1	1268.5	0.157	0.13
Among populations within groups	4	405,584.5	502.0	0.062	0.01
Within populations	611	7,277,053.1	5609.8	0.695	0.01
Within genotypes	617	426,017.5	690.5	0.086	0.01

**Table 2:** Genetic differentiation of the inferred populations of modern German winter wheat and the A.E. Watkins landrace core collection. Means of the polymorphism information content (PIC), of the minor allele frequency (MAF), of the inbreeding coefficient *F*_*IS*_, of the observed heterozygosity (Ho), and the expected heterozygosity (He). The four German winter wheat (GWW) breeding programs are abbreviated GWW1–GWW4

	PIC	MAF	F	H_o_	H_e_	N
GWW1	0.25	0.24	0.83	0.05	0.32	174
GWW2	0.24	0.22	0.87	0.04	0.29	173
GWW3	0.25	0.23	0.85	0.05	0.31	90
GWW4	0.22	0.20	0.96	0.01	0.28	31
Varieties	0.26	0.24	0.99	0.00	0.32	30
Elite	0.26	0.242	0.985	0.005	0.37	498
Elite (50 × random, n = 119)	0.26	0.24	0.96	0.01	0.37	119
Watkins	0.29	0.28	0.99	0.00	0.37	119
All sets	0.29	0.27	0.91	0.03	0.36	617

### Elite winter wheat germplasms reveal pronounced genetic variation in agronomic and quality traits

In addition to molecular genetic diversity, we were also interested in the variation of phenotypic traits. Therefore, the set of European elite wheat inbreds has been evaluated over two years in 14 contrasting environments for four agronomic and quality traits including grain yield (GYD), plant height (PHT), heading date (HDT), and grain protein content (GPC). An analysis of variance (ANOVA) revealed that both genotypic and genotype-by-environment (GxE) variances were statistically significant (*p* < 0.001) for all traits in and across both years (Table 3). Moderate-to-high heritability estimates depict a low error variance and a high genetic variance for all traits. The frequency distribution for all phenotypes indicated that normal distribution is a reasonably good approximation (Supplementary Fig. 2). Noteworthy, the GxE interaction variances of GYD were greater than the variances of the genotype under the drought stress condition in 2022 (Table 3).

**Table 3:** Estimation of genetic parameters, variance components, and broad-sense heritability of German winter wheat genotypes evaluated in 2021 and 2022 cropping seasons. Genotype, σ^2^_G_; genotype-by-environment interaction, σ^2^_GxE_; error, σ^2^_e_) and H^2^, heritability; GYD [Mg ha^−1^], grain yield in mega gram per hectare; PHT, plant height in centimeters; HDT, heading date (days after sowing); and GPC, grain protein content % volume per seed dry weight

Year	2021						2022					
Trait	μ ± s	x_min_-x_max_	σ^2^_G_	σ^2^_GxE_	σ^2^_e_	H^2^	μ ± s	x_min_-x_max_	σ^2^_G_	σ^2^_GxE_	σ^2^_e_	H^2^
GYD [Mg ha^−1^]	8.185 ± 3.97	6.92—9.21	1.28^***^	1.23^***^	1.75	0.81	7.08 ± 0.31	6.18 – 8.03	0.71^***^	0.84^***^	1.65	0.74
PHT [cm]	88.06 ± 5.95	73.88–110.62	33.22^***^	3.68^***^	12.87	0.94	82.61 ± 5.29	69.70–97.49	25.26^***^	6.1^***^	10.3	0.91
HDT [days]	39.09 ± 1.68	34.44–43.14	2.67^***^	0.38^***^	0.67	0.94	28.29 ± 1.64	23.88–32.90	2.5^***^	0.66^***^	0.38	0.94
GPC [%]	11.50 ± 0.44	10.04–12.83	0.14^***^	0.15^***^	0.31	0.73	11.51 ± 0.45	10.44 -12.98	0.16^***^	0.13^***^	0.15	0.86

### Level of drought stress and its effect on mean trait values

**Fig. 2:**
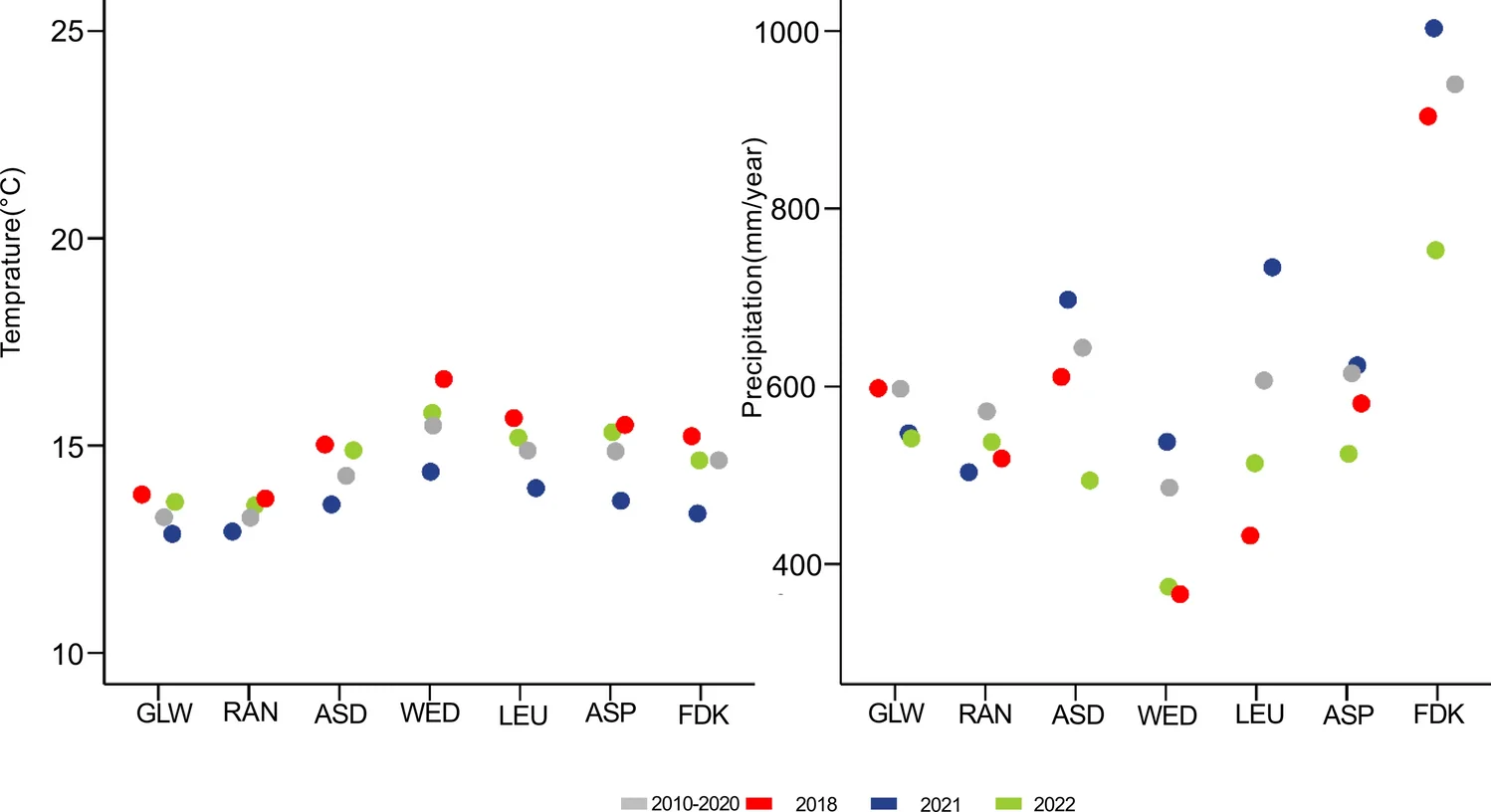
Annual mean maximum temperature (**A**) and total precipitation (**B**) of the seven test locations in year 2010–2020 (long-term average), 2018 (severe drought), 2021, and 2022 (cropping seasons). Color codes in B are the same as in A. The cropping season is from September in the previous year to August of the actual year. Ranzin, RAN; Aspachhof, ASP; Groß Lüsewitz, GLW; Leutewitz, LEU; Weddegast, WED; Asendorf, ASD; and Feldkirchen, FDK

**Fig. 3:**
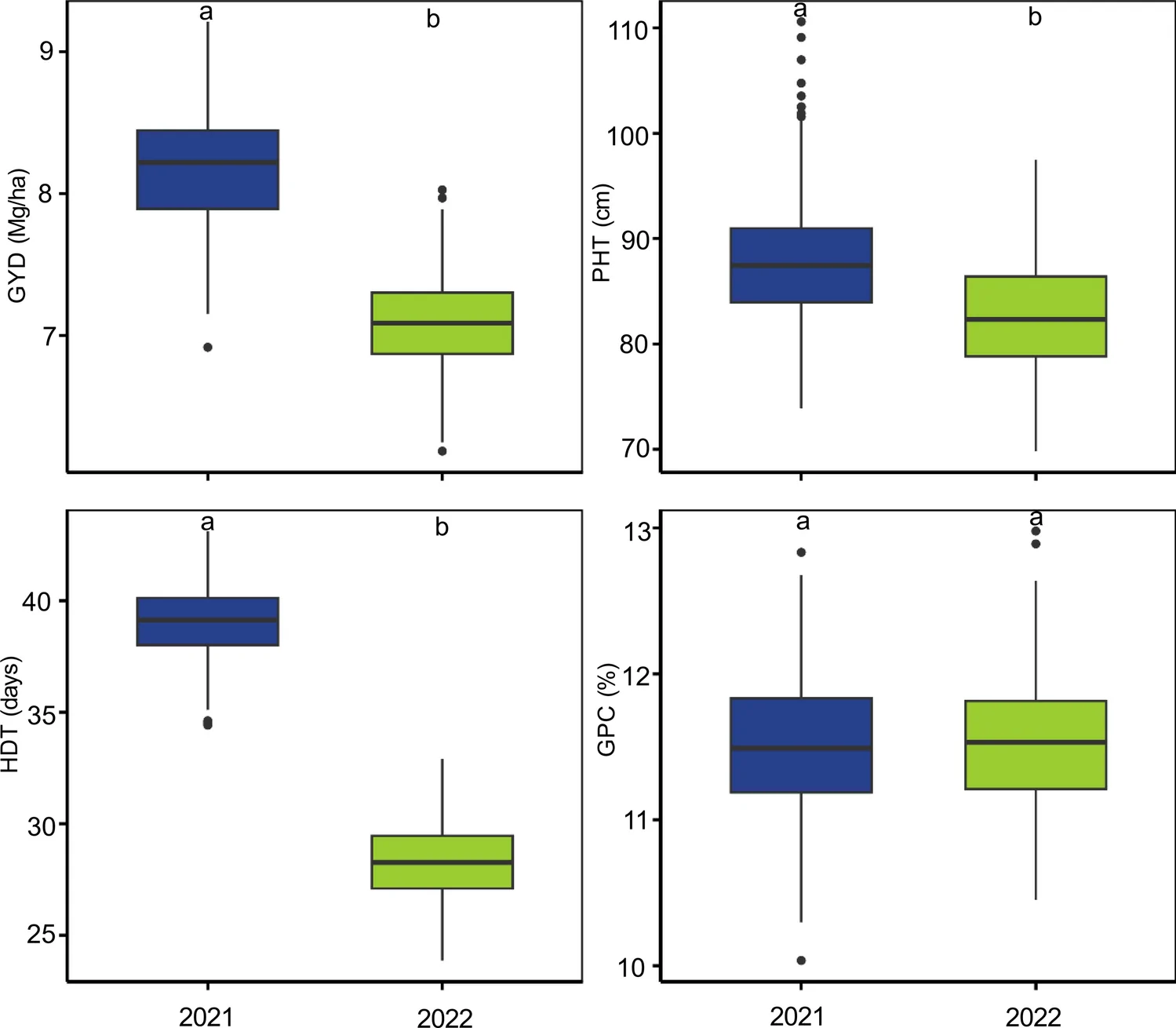
Boxplots of the best linear unbiased estimates (BLUEs) for four traits of the German winter wheat panel measured under two contrasting years for moisture stress under rain-fed conditions in 2021 and 2022 cropping seasons. GYD, grain yield; PHT, plant height; HDT, heading date; and GPC, grain protein content. Different letters indicate significant differences based on a two-tailed Tukey test (*p* < 0.001)

The mean annual temperature and precipitation recorded for the test locations revealed distinct temperature deviations across environments compared to the long-term average and the extreme drought year of 2018, reflecting both inter-annual variability and regional climatic differences (Fig. 2A and B). In 2021, mean temperatures and precipitation were generally close to the long-term average, with some environments exhibiting minor deviations. In contrast, the year 2022 showed a consistent increase in mean temperature across all environments without reaching the extreme conditions observed in 2018. However, most 2022 environments revealed an even lower precipitation as compared to 2018. This drought stress caused a significant drop in the performance of genotypes, resulting in an average GYD loss of 13.7% in the 2022 cropping season compared to 2021 (Fig. 3). In comparison with the non-stress conditions 2021, we observed also a decline in PHT and HDT under the drought stress 2022. In contrast, the difference in GPC between the two cropping seasons was minimal.

### Effect of the wheat–rye translocations on grain yield and grain protein content under drought

**Fig. 4:**
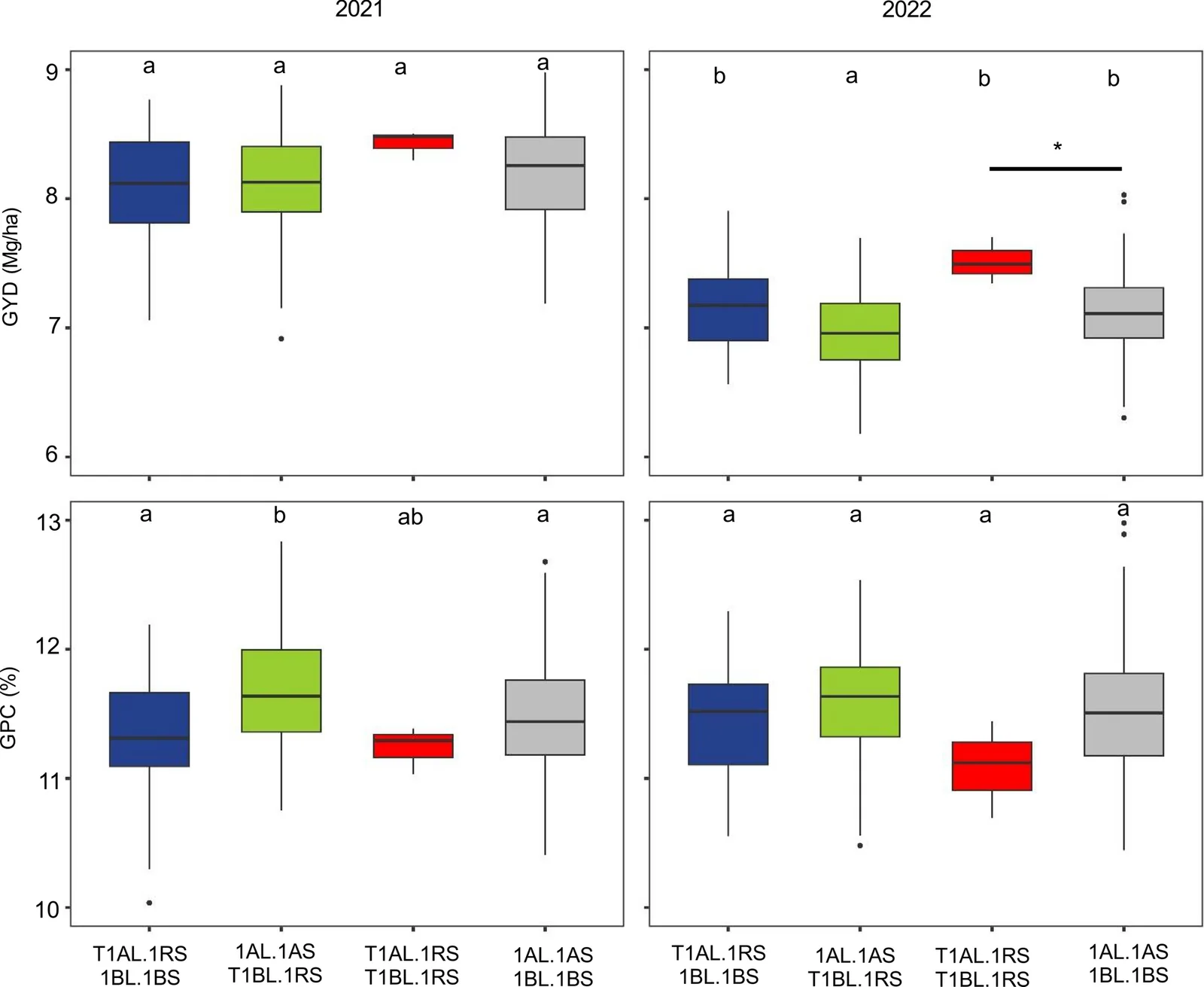
The effect of wheat–rye translocations on grain yield (GYD) and grain protein content (GPC) for each cropping season 2021 and 2022. Grain yield (GYD)—upper row, grain protein content (GPC)—lower row. Different letters indicate significant differences based on a two-tailed Tukey test (*p* < 0.05). The asterisk (*) marks additional significant (*p* < 0.05) pairwise differences between groups. The boxes show the range from first to third quartiles divided by the median. The whiskers span from the minimum to the maximum observations. T1AL.1RS (1RS is translocated on the long arm of wheat chromosome 1A designated as 1AL), T1BL.1RS (1RS is translocated on the long arm of wheat chromosome 1B designated as 1BL), T1AL.1RS/T1BL.1RS (genotypes with both translocations: T1AL.1RS and T1BL.1RS), and 1AL.1AS/1BL.1BS (without the 1RS translocations). 1RS, the short arm of rye chromosome 1R

We were particularly interested in the performance of the different rye translocations across the diverse environmental conditions. Except for GYD in 2021 and GPC in 2022, we detected significant (*p* < 0.001) differences among the four genotype groups defined according to their 1RS translocation status (Fig. 4). Grain yield performance under drought was significantly (*p* < 0.001) lower in T1BL.1RS translocation wheat than in the other genetic groups (Fig. 4B). Group-wise stability variances are shown in Supplementary Fig. 3. The T1BL.1RS group exhibited the lowest stability variance, whereas T1AL.1RS and the stacked T1AL.1RS/T1BL.1RS group showed higher values (Supplementary Fig. 3). Because T1BL.1RS genotypes were also the lowest-yielding under drought, this lower variance reflects stable performance at a reduced yield level rather than superior resilience.

An independent t test indicated that the genotype group with double translocation (T1AL.1RS/T1BL.1RS) had a significantly (*p* < 0.05) higher GYD under drought than the genotype group without the wheat–rye translocation (1AL.1AS/1BL.1BS), highlighting the yield advantage of the double translocation over the non-translocated genotype group (Fig. 4B). This specific pairwise difference is highlighted by an asterisk in the figure, whereas letter groupings represent multiple comparisons derived from the overall ANOVA. Across both seasons, the double translocation carriers tended to express the lowest GPC values, consistent with the expected dilution effect of the stacked translocation. However, despite the intricate trade-offs between grain yield and grain protein content, we did not detect significant differences among the genotype groups for GPC under drought (Fig. 4B).

### Impact of the short arm of rye chromosome 1R on yield performance and stability in winter wheat

**Fig. 5:**
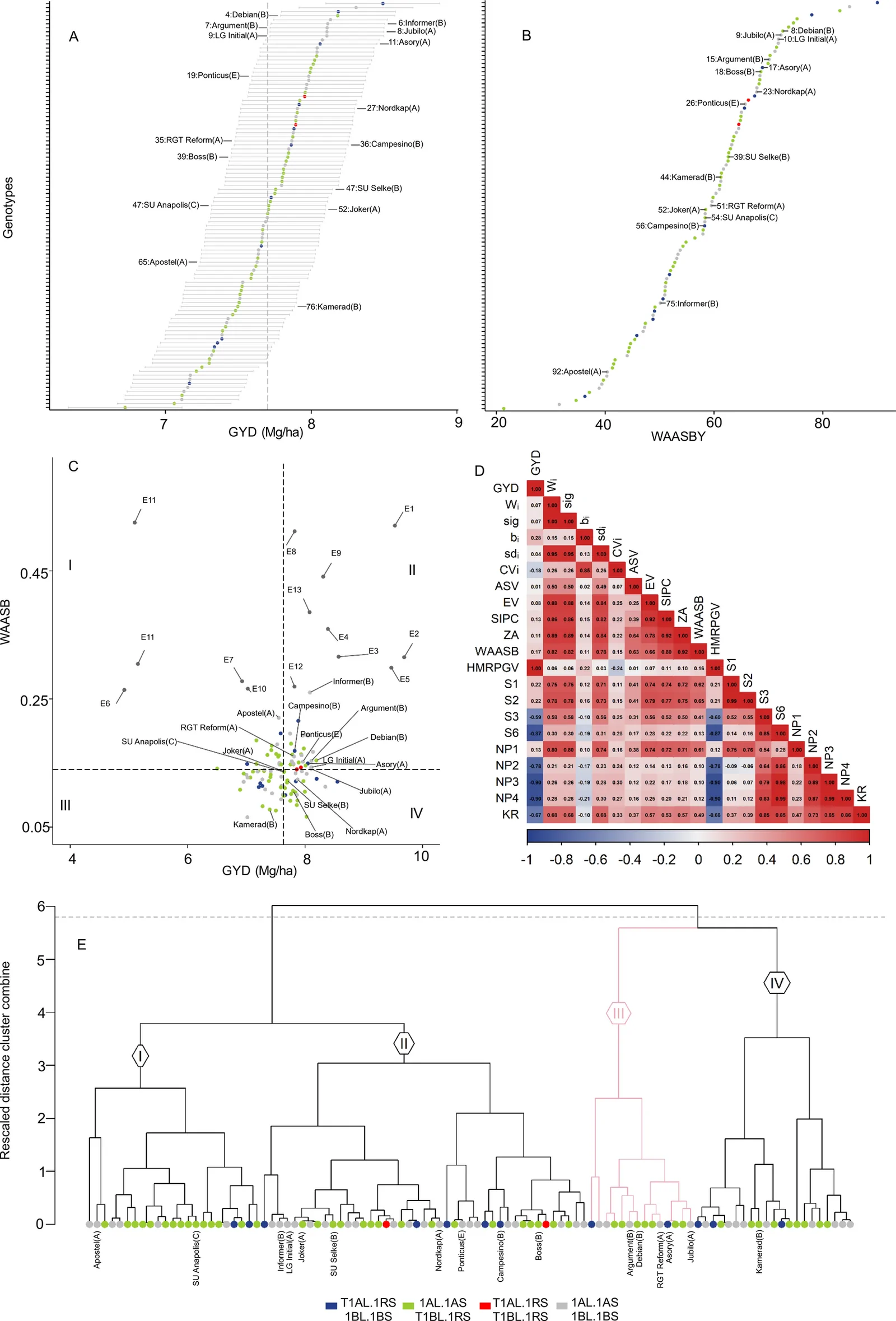
(**A**) The best linear unbiased prediction (BLUP) of 101 winter wheat genotypes for grain yield (GYD Mg ha-1) from 14 environments (E1 to E14). The vertical dotted line indicates the grand mean, indicating genotype performance above or below the grand mean; horizontal error bars indicate the 95% confidence interval from a two-tailed t test. (**B**) Estimated values of the weighted average of the stability (WAASB) and mean grain yield (WAASBY). (**C**) Biplot of mean grain yield (GYD Mg ha-1) and the weighted average of absolute scores for the BLUPs of the GEI WAASB. (**D**) Spearman’s correlation coefficients among ranks of yield, parametric and nonparametric stability statistics for 101 winter wheat genotypes tested across 14 environments. *, **, *** indicates significant at the 0.05, 0.01, and 0.001 probability level, respectively. (**E**) Dendrogram showing the hierarchical classification of 101 winter wheat genotypes based on ranks of mean GYD and the average sum of ranks of all stability statistics (ASR). The black dashed horizontal line is cutoff line. The WAASBY was computed considering the weights of 65 and 35 for yielding and stability, respectively. Color codes indicate genetic groups. T1AL.1RS (1RS is translocated on the long arm of wheat chromosome 1A designated as 1AL), T1BL.1RS (1RS is translocated on the long arm of wheat chromosome 1B designated as 1BL), T1AL.1RS/T1BL.1RS (genotypes with both translocations: T1AL.1RS and T1BL.1RS), and 1AL.1AS/1BL.1BS (without the 1RS translocations). 1RS, the short arm of rye chromosome 1R

A highly significant genotype × environment interaction for grain yield was detected in the AMMI model (*p* < 0.001; Supplementary Table S2). To evaluate genotypic performance across environments in terms of both yield and stability, we focused on a subset of 101 winter wheat genotypes that were consistently evaluated in both years. This subset comprised 40 non-translocation lines, 12 T1AL.1RS, 47 T1BL.1RS, and 2 stacked T1AL.1RS/T1BL.1RS genotypes and formed the basis for all downstream GEI and performance stability analyses (Supplementary Tables 3 and 4). We first estimated best linear unbiased predictors (BLUPs) to assess the expected GYD performance of these 101genotypes (Fig. 5A, Supplementary Table 3). Among the top ten genotypes, three carried a rye translocation, two with T1AL.1RS and one with T1BL.1RS, while the remaining seven were non-translocation lines, indicating that genetic groups contributed to high yield performance across contrasting environments. Five of the top ten genotypes were released cultivars, including three milling wheats and two bread wheats. Notably, two of the top three ranked genotypes (1st and 3rd) belonged to the T1AL.1RS translocation group. The bread wheat cultivar ‘Asory,’ also carrying T1AL.1RS, maintained competitive drought performance relative to current elite breeding lines, consistent with its reported superior yield under extreme drought conditions in 2018.

The distribution of genotypes carrying the 1RS translocation across the full yield spectrum demonstrates that the presence of the translocation itself does not confer a consistent performance advantage. This result implies that it is not the 1RS translocation per se, but the specific combination of background and translocation that determines agronomic performance. We tested for a translocation-by-genetic background interaction affecting grain yield by comparing two genomic selection (GS) models for grain yield based on our entire wheat panel set. Model 1 included only the main effects of the 1RS translocation and the polygenic background, while Model 2 also included their interaction. A likelihood ratio test showed that including the interaction significantly (*p* < 0.01) improved model fit (Supplementary Table 4), confirming the translocation-by-background effect on grain yield. Furthermore, variance component estimates from the genomic model indicated that the majority of the phenotypic variance for grain yield was explained by the polygenic background (σ^2^_G_ ≈ 83%). The SNP × 1RS interaction (σ^2^_G × T_) accounted for a smaller but statistically significant proportion (≈ 5.7%), while residual variance contributed ≈ 11.4% (Supplementary Table S9). These results quantitatively confirm that the effect of the 1RS translocation on yield is partially dependent on the genetic background and moderate in magnitude.

To jointly evaluate mean GYD and stability, we applied the weighted average of absolute scores (WAASB) based on BLUP-derived genotype × environment interaction effects. WAASB integrates yield performance and stability by decomposing GEI into principal components, thereby providing a balanced measure of adaptability across environments. Incorporating WAASB reshaped genotype rankings compared to mean yield alone (Fig. 5B). Several T1BL.1RS lines showed marked rank improvements when stability was considered, while a T1AL.1RS line maintained a top position across both metrics. These results indicate that certain 1RS configurations combine high yield potential with stable performance across contrasting environments (Supplementary Table 6).

The WAASB biplot (Fig. 5C) separated genotypes into four quadrants representing distinct yield stability combinations. A higher proportion of 1RS genotypes was observed among entries combining above-average yield and stability (76%) compared to the overall panel frequency (60.4%), suggesting an enrichment of favorable performance patterns within specific translocation backgrounds.

To assess robustness of stability patterns, we additionally evaluated parametric and nonparametric stability indices (Supplementary Table 5). Spearman’s rank correlation revealed predominantly positive associations among most stability measures, indicating broad concordance across indices (Fig. 5D; Supplementary Fig. 3). Several of these correlations were statistically supported (*p* < 0.001), including strong agreement with Wricke’s ecovalence (Wi). Differences in genotype rankings (e.g., ASR vs. WAASBY; Fig. 5B) illustrate the complementary nature of stability metrics rather than the superiority of any single index. In contrast, Thennarasu’s NP3 displayed a divergent correlation pattern.

While correlations were primarily calculated based on trait means across environments to capture overall relationships among genotypes, environment-specific correlations may provide additional insights into genotype-by-environment interactions. Nevertheless, the consistency of the observed relationships across environments supports the robustness of conclusions drawn from aggregated data.

Overall, clustering patterns reflected pronounced response diversity within the panel and demonstrated that both broad and specific adaptability can be targeted in modern winter wheat breeding.

Hierarchical clustering analysis (HCA) based on mean GYD and the average sum of ranks (ASR) grouped genotypes into four clusters with distinct yield stability profiles (Fig. 5E, Supplementary Table 7). Cluster III combined high yields (8.06 Mg ha⁻^1^ vs. average 7.63 Mg ha⁻^1^) with low stability variance and contained an elevated proportion of 1RS genotypes relative to the panel mean. Cluster II contained high-yielding but less stable entries, Cluster IV combined moderate yield with high stability, and Cluster I low-yielding and unstable entries. Overall, clustering underscores pronounced response diversity within the panel and demonstrates that both broad and specific adaptability can be targeted in modern winter wheat breeding.

### Selection response and the adaptive significance of 1RS translocations

We investigated the response of German winter wheat genotypes, including 30 released varieties with different baking qualities, to selection under contrasting natural precipitation patterns. Initially, we determined the selection differential (ΔS_GYD_) in relation to the whole population by identifying the top 10% of wheat lines for GYD in each year (Supplementary Table 8). Selection led to a positive ΔS_GYD_ under both precipitation scenarios, improving yield potential by 7.8% in 2021 and 7.2% in 2022. This demonstrates the significant genetic and phenotypic diversity in wheat production systems. We observed that only four of 30 and 31 genotypes overlapped between the chosen groups in 2021 and 2022. Furthermore, we observed differences in flowering time (26–31 days) and plant height (74–92 cm) among the selected lines (Fig. 3).

**Fig. 6:**
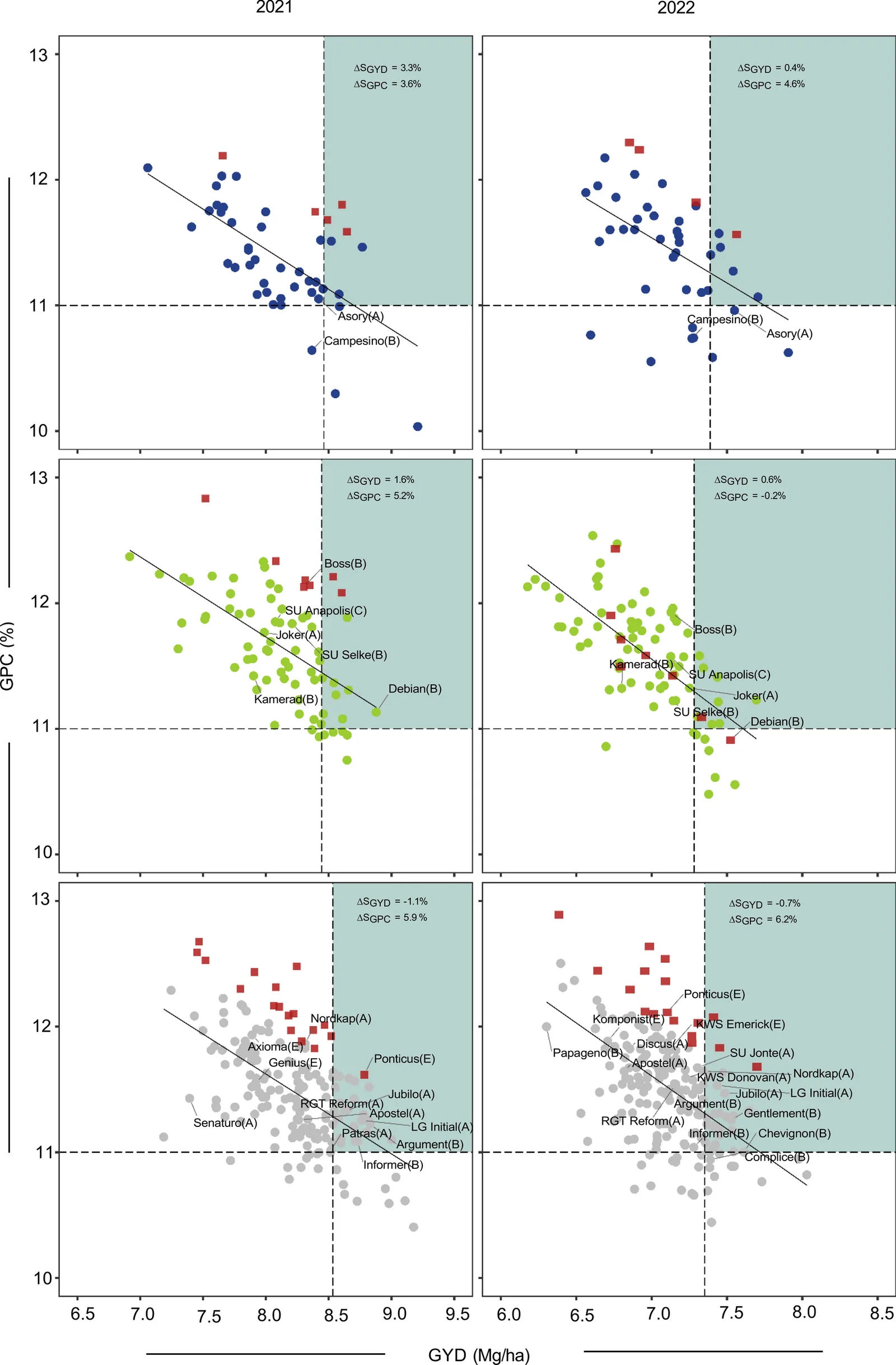
Scatter plots of the correlation between grain yield (GYD) and grain protein content (GPC) in the three genetic groups in two cropping seasons (2021–2022). Blue, green, and gray codes indicate genotypes from a genetic group T1AL.1RS (1RS is translocated on the long arm of wheat chromosome 1A designated as 1AL), T1BL.1RS (1RS is translocated on the long arm of wheat chromosome 1B designated as 1BL), and 1AL.1AS/1BL.1BS (without the 1RS translocations), respectively. The letter ‘T’ indicates the presence of the translocation. 1RS (short arm of rye chromosome 1R). The solid line is the regression line of grain yield on protein content. For reference, some important varieties with and without translocations are indicated. Letters in brackets indicate the wheat quality classes of the German wheat classification system grades according to their baking quality. The horizontal and vertical dashed lines indicate a threshold of 11% GPC and the 20% highest yielding wheat lines, respectively. The red square symbols mark the 10% best genotypes according to phenotypic index PDV in the respective genetic groups. The shaded quadrant marks genotypes with high GYD and GPC. Selection differentials for GYD and GPC content were calculated as the difference between the mean of the 10% best-performing genotypes of the genetic groups and the mean of the base genetic group population (ΔS)

Second, we used the grain protein deviation (PDV) as selection index to evaluate whether favorable combinations of grain yield (GYD) and grain protein content (GPC) could be identified within each genetic group over a two-year period, despite the commonly observed trade-off in wheat (Fig. 6). For comparison, results obtained with alternative indices (YDV, EPY, EWD) are summarized in Supplementary Table 9. We selected genotypes within the top 10% for PDV that exceeded the GYD grand mean and an 11% GPC threshold, reflecting the minimum protein level considered relevant for Central European bread-making quality; genotypes in the feed/cookie segment were therefore not targeted. In the selected fraction, non-translocation lines displayed negative selection differentials for GYD (–1.1% in 2021, –0.7% in 2022) but positive differentials for GPC. In contrast, both the T1AL.1RS and T1BL.1RS groups showed positive selection differentials for GYD, accompanied by only minor negative selection differential for GPC (-0.2%, T1BL.1RS). Under well-watered conditions, the T1AL.1RS group exhibited the most balanced selection response, indicating a partial mitigation of the yield–protein trade-off rather than a complete decoupling of both traits.

## Discussion

In this study, we conducted a comprehensive assessment of genotypic and phenotypic diversity in modern German winter wheat with a particular emphasis on the impact of rye translocations under increasingly variable environmental conditions as well as a comparison to the genetic diversity of the A.E. Watkins landrace core collection.

### Elite German winter wheats retain substantial genetic diversity

Drought stress in the 2022 cropping season caused an average reduction of 13.7% in grain yield compared to 2021, highlighting the severity of climate-induced variability in Central European wheat production. The substantial genotypic variance in yield and agronomic traits (Table 3) observed under the near-optimal precipitation conditions of 2021 (comparable to 1961–1990 averages) indicates that high yield potential is still present in current German winter wheat breeding germplasm, i.e., in material that is younger and more diverse than the sets typically analyzed in national variety trials. This underlines the need to evaluate such breeding material across both favorable and stress-prone environments. Concerns have been raised about the homogenization of the wheat genetic pool and the loss of alleles through modern breeding practices (Kahiluoto et al. 2019; Bapela et al. 2022). In this context, our diversity analysis revealed a similar level of diversity in German elite winter wheats compared to the A.E. Watkins landrace core collection which was assembled as a reference representing global genetic diversity of wheat (Fig. 1, Table 2). These results indicate that the applied SNP array captures a non-negligible proportion of allelic variation within the current German elite breeding material, despite elite × elite crosses and decades of directional selection in modern wheat breeding (Sharma et al. 2021). However, it is important to note that SNP arrays are typically ascertained in elite breeding material, which may influence relative diversity estimates when comparing elite lines with landrace collections. Consequently, similarity in diversity metrics should not be interpreted as equivalence in overall allelic richness or evolutionary diversity. Such estimates reflect array-based diversity metrics and do not necessarily capture differences in overall allelic richness, rare alleles, or broader evolutionary diversity between elite germplasm and landrace collections.

The observed level of expected heterozygosity in German elite winter wheat approximates the genetic diversity seen in synthetic hexaploid wheat (Bhatta et al. 2018) and even exceeds that reported for a panel of ten US bread wheat cultivars. While the latter comparison highlights the considerable diversity retained within German breeding programs, it should be interpreted with caution, given the limited size and scope of the US panel. Moreover, the two countries differ significantly in wheat production intensity and breeding priorities, with Germany focusing predominantly on high-input, high-yielding systems in temperate environments, while US programs often operate under broader agroecological zones. These differences likely influence the genetic architecture within each breeding system and make our observation even more surprising. Our finding that German elite winter wheat matches or exceeds the diversity levels reported for other major germplasm pools (Eltaher et al. 2018; Türkoğlu et al. 2023; Liu et al. 2024) underscores the effectiveness of German breeding programs in preserving adaptive potential under increasing climate variability. This is presumably because these programs have systematically managed and monitored genetic diversity, particularly with respect to alleles of agronomic and quality relevance, as well as disease resistance. Our results underscore the value of systematic crosses between diverse parental material in elite winter wheat breeding, the continuous introduction of novel alleles including targeted introgressions from gene banks and wild relatives, particularly for resistance loci, and the effectiveness of recombination. This pattern indicates that systematic crossing and recombination strategies have maintained substantial adaptive variation at the elite level in breeding programs.

The low but obvious population structure observed among elite wheats is consistent with the results of Lell et al. (2025). Given wheat’s autogamous nature, the absence of population structure reflects systematic crossing efforts, and effective recombination.

Compared to recent whole-genome resequencing analyses of the Watkins landraces (Fig. 1) (Cheng et al. 2024), the weaker population structure observed in our study likely results from ascertainment bias inherent to the SNP array, which was primarily developed using elite germplasm. Consequently, diversity patterns captured here represent variation at common polymorphic loci included on the array rather than comprehensive genome-wide allelic richness. Similar limitations of breeder-oriented SNP arrays in representing rare alleles and broader historical diversity have been reported previously (Allen et al. 2017; Burridge et al. 2024). Nonetheless, key diversity metrics validate the utility of the array for both modern and traditional germplasm. Principal coordinate analysis revealed a clear genetic separation between the elite German winter wheat genotypes and the Watkins landrace collection. The elite genotypes formed a tight cluster along the first principal coordinate, reflecting their shared breeding history. In contrast, the Watkins accessions were more broadly distributed and structured into seven distinct ancestral groups (AGs), with AG2 partially overlapping the elite germplasm. This pattern highlights the Watkins collection as a valuable reservoir of allelic diversity. The pronounced divergence between elite and landrace germplasm reinforces the potential of landraces to broaden the genetic base and introduce novel variation into winter wheat breeding programs. Notably, the observed overlap between elite wheat and Watkins AG2 (Fig. 1) aligns with findings from Cheng et al. (2024), supporting the hypothesis that this ancestral group contributed to the founder lines of modern wheat.

### 1RS translocations are common in German winter wheat

The historical relevance of rye translocations in boosting stress tolerance and yield is well described (Han et al. 2025). However, concerns about quality penalties and other linkage drag effects have cast doubt on their value and made our study necessary.

Our study revealed the widespread (21.7 and 14.6%) presence of both 1RS translocations in elite German winter wheat (Fig. 1, Supplementary Table 1). This prevalence likely reflects the historical breeding value attributed to T1BL.1RS, particularly in relation to disease resistance and agronomic robustness, as reported in previous studies. Recent efforts to refine rye introgressions further indicate their continued relevance for modern breeding objectives. The frequency of 1RS translocations in elite German winter wheat was lower compared to that in Chinese material as summarized by Han et al. (2025). Nevertheless, our findings confirm the systematic integration of both T1AL.1RS and T1BL.1RS, with distinct patterns and implications for German wheat breeding.

### T1AL.1RS and T1BL.1RS have distinct agronomic and grain quality profiles

One of the striking findings of our study was the persistence of T1BL.1RS, despite its significantly poorer drought performance. Its continued use is likely driven by its consistently high GPC under optimal environmental conditions, a trait that remains a major selection criterion in German wheat breeding, and is widely used as a proxy for baking. In contrast, the T1AL.1RS group exhibited a more balanced selection differential for both GYD and GPC, particularly under optimal moisture conditions (Fig. 6). This pattern differed from other groups, in which selection responses tended to favor one trait over the other. Importantly, this observation does not negate the general yield–protein trade-off across genotypes but indicates that T1AL.1RS lines can achieve a comparatively favorable trait combination under specific environmental conditions. The differences reflect the distinct functional impact of losing either 1AS or 1BS, with the loss of the 1AS arm in T1AL.1RS being less detrimental to end-use quality than the loss of 1BS in T1BL.1RS (Graybosch et al. 1993). These distinctions are mirrored in the performance of cultivars ‘Asory’ (T1AL.1RS) and ‘Kamerad’ (T1BL.1RS), both tested under severe drought in the 2018 German national cultivar trials (Rabanus-Wallace et al. 2021), which we use here as reference checks, and again observed in the present study in 2022.

Breeders appear to have managed the trade-offs associated with both translocations through careful selection, recombination, and genotype-specific adaptation, re-contextualizing 1RS not as a binary trait with fixed outcomes but as a nuanced breeding tool. Compared to the exploitation of all seven rye chromosomes in triticale, targeted introgressions such as 1RS translocations offer greater precision and efficiency. While triticale excels under abiotic stress, its adoption remains limited by agronomic constraints and lower end-use quality. In contrast, the controlled integration of rye chromatin into wheat preserves essential quality traits while leveraging rye’s adaptive potential (Hackauf et al. 2022), a strategy better suited for scalable, climate-resilient wheat breeding.

### Double 1RS translocations confer drought resilience in wheat

Recent studies underscore the significant economic impact of the 2022 drought that affected Europe, with crop production losses estimated to €13 billion for major arable crops and up to €25–30 billion when including all croplands (Pinke et al. 2024). This macroeconomic backdrop highlights the urgent need for breeding strategies that enhance crop resilience to increasingly frequent and severe abiotic stresses. In Germany, yield losses due to combined heat and drought stress varied significantly by site, with the highest losses observed at locations characterized by limited water-holding capacity and reduced buffering against extremes, features typically found in regions with sandy soils and lower precipitation (Riedesel et al. 2024a). These spatial patterns support our multi-environment field-phenotyping approach, which was designed to capture genotype × environment interactions that are essential for selecting stable, climate-adapted elite lines. As emphasized by Abraham Blum’s call for delivering solutions to farmers (Blum 2011), the success of drought resistance breeding depends on identifying the right physiological traits under the relevant field conditions. Our design therefore reflects two guiding principles emphasized in wheat drought breeding: (i) Genotypes must be evaluated under the relevant stress environments (Semahegn et al. 2020; Langridge and Reynolds 2021), and (ii) drought adaptation should be addressed as a multi-trait syndrome rather than as a single-trait target, where breeding progress depends on capturing these interacting traits under the target stress environment (Langridge and Reynolds 2021; Bapela et al. 2022).

Despite sustained yield improvements in German wheat breeding, long-term analyses reveal increasing yield losses under heat and drought stress and a lack of improved tolerance in newer wheat cultivars (Laidig et al. 2021; Riedesel et al. 2024b). While previous studies focused on released varieties, our results provide deeper insight into the diversity actively created and maintained within modern breeding programs, providing the critical resource for developing climate-adapted wheat. The identification of elite breeding lines outperforming released cultivars under severe natural drought conditions as in 2022 underscores the potential of harnessing genetic variation and rye introgressions to enhance stress resilience and yield stability.

One of the tools to achieve this is the targeted exploitation of 1RS translocations. Genotypes carrying both T1AL.1RS and T1BL.1RS performed superior under drought, suggesting additive or potentially synergistic effects of stacked rye segments. In view of previously reported effects of the T1AL.1RS translocation on the root biomass and root system architecture (Waines and Ehdaie 2007), the observed advantage may be associated with altered root development and stress responsiveness. However, root traits and physiological parameters were not directly assessed in the present study. Therefore, any mechanistic interpretation remains hypothetical and requires dedicated phenotypic and functional validation in the genotypes analyzed here. The consistently lower grain protein concentration observed for the double 1RS carriers across both seasons may also influence end-use quality, as reduced GPC can compromise baking performance if not balanced by strong gluten characteristics. This underlines the need of integrating grain quality selection when deploying stacked 1RS configurations for resilience breeding. Notably, the allocation of released cultivars, carrying no 1RS translocation and used as a reference, to adjusted stability ranking (ASR)-based cluster III (Fig. 5C, 5E) supported the view that current breeding strategies are successfully accounting for climate variability. This finding also indicated that 1RS should be viewed as a contributor to stress adaptation of modern winter wheat. By enhancing yield stability under adverse conditions, such traits help safeguard the productivity base of cereal systems. This is particularly relevant as climate change-induced yield losses hamper the greenhouse gas mitigation potential of German cereal production, particularly in key wheat-growing regions (Riedesel et al. 2024a). Thus, breeding for drought and heat resilience not only secures food production but also supports national climate mitigation targets by maintaining the carbon efficiency of domestic cereals.

### T1AL.1RS partially mitigates the yield-protein trade-off

The well-documented negative correlation between grain yield and protein content in wheat presents a persistent breeding challenge (Richard et al. 2025). In our study, several genotypes deviated from this general trend, indicating that favorable yield–protein combinations can be identified under specific genetic and environmental contexts.

In contrast to non-translocation lines, which showed negative and positive selection differentials for yield and protein content, respectively, T1AL.1RS lines exhibited positive selection differentials for both grain yield and protein content across years. This results suggest a partial mitigation of the classical yield-protein trade-off rather than a complete decoupling of both traits. A similar pattern of positive selection differentials was observed for the T1BL.1RS translocation under non-stressed conditions. The observed performance of T1AL.1RS may reflect genotype-specific effects on resource allocation or stress adaptation; however, the underlying physiological mechanisms were not directly assessed in this study. While T1BL.1RS carriers did not consistently outperform T1AL.1RS or stacked translocation genotypes under drought, their relatively stable protein performance under optimal conditions and yield stability, as reflected by WAASB-based stability assessment and the ASR-supported clustering (Fig. 5C–E) likely explain their continued use in elite breeding materials. This is consistent with national breeding priorities that emphasize bread-making quality. Recent work by Nagel-Held et al. (2024) further illustrates that grain protein content alone does not fully predict baking performance, highlighting the importance of context-specific quality assessment. Together, our results support a differentiated view of 1RS introgressions, in which specific configurations may contribute to improved performance depending on environmental conditions and recipient background.

### Genetic background influences 1RS expression

The distribution of genotypes carrying the 1RS translocation across the full yield spectrum demonstrated that the presence of the translocation does not confer a consistent performance advantage per se but illustrates the relevance of the genetic background (Fig. 5). Furthermore, our analyses (Supplementary Table 4) supported a translocation-by-polygenic background effect on grain yield. These results are in line with previous studies that reported both positive and negative agronomic effects of the 1RS translocation, indicating that its breeding value is strongly influenced by genetic background and environmental conditions (Ehdaie et al. 2003; Sharma et al. 2010; Mathews et al. 2008; Pinto et al. 2010; Peake et al. 2011). Our results provide quantitative support for this interpretation. Variance component estimates from the genomic model showed that the polygenic background explained the majority of phenotypic variance in grain yield (≈83%), whereas the SNP × 1RS interaction accounted for a smaller but statistically significant proportion (≈5.7%). Although moderate in magnitude, this interaction component indicates that the agronomic effect of 1RS cannot be considered uniform across genetic backgrounds. To our knowledge, this study represents one of the first quantitative assessments of how the polygenic wheat background modulates the agronomic expression of 1RS introgressions in modern elite germplasm evaluated across multiple environments. Importantly, the predominance of the polygenic background in explaining yield variation indicates that modern elite germplasm still contains substantial recombined diversity that can be exploited for climate resilience, with 1RS introgressions acting as context-dependent modifiers rather than universal yield enhancers. Evidence for such context-dependent effects has also been observed in large breeding datasets. For example, analyses of the US Department of Agriculture (USDA) Regional Performance Nursery showed that the T1RS.1BL translocation was associated with an average yield increase of 4.55%, while the T1RS.1AL translocation conferred a smaller but still significant 0.86% improvement (Rabanus-Wallace et al. 2021). Conversely, the Kansas State University wheat panel reported that T1RS.1AL delivered a more substantial yield advantage of 4.06%, outperforming T1RS.1BL, which showed only a 1.50% increase (Han et al. 2025). These contrasting observations likely reflect epistatic interaction between the translocation and the recipient wheat background as well as genotype-by-environment interactions affecting trait expression. From a breeding perspective, such context-dependent effects highlight the importance of considering genetic background when deploying 1RS introgressions. Breeding strategies and genomic prediction models should therefore account for these interactions rather than assuming uniform benefits from the presence of 1RS.

### Multi-environment trials highlight the effect of translocations on stability and adaptation

The strong influence of the polygenic background on the expression of 1RS introgressions also has implications for how their agronomic effects should be interpreted across environments. If the contribution of rye chromatin is shaped by interactions with the recipient genome and environmental conditions, its effects are most clearly revealed in multi-environment analyses that capture genotype-by-environment interactions. In this context, stability metrics provide a useful framework for identifying introgressions that contribute to consistent performance across diverse environments. Notably, some advantages of specific 1RS configurations were not apparent from mean performance alone but emerged when yield was interpreted jointly with stability metrics (WAASBY) and clustering-based profiles. Our multi-environment trials analyzed using WAASB-based statistics therefore provide a complementary perspective on the agronomic relevance of 1RS introgressions. Notably, genotypes carrying 1RS translocations were overrepresented (21%) among high-yielding and stable genotypes (quadrant IV) relative to their overall frequency in the panel (Fig. 5). This enrichment of 1RS carriers among high-performing and stable genotypes further supports the interpretation that the contribution of rye chromatin is expressed in a background- and environment-dependent manner rather than as a uniform yield advantage. The WAASB index showed broad concordance with classical stability parameters while remaining largely independent of mean yield (Fig. 5D), supporting its suitability as a GEI-focused stability descriptor (Reckling et al. 2021). An alternative strategy to increase yield stability in wheat breeding is the development of hybrid cultivars. Mühleisen et al. (2014) illustrated the potential of this approach. In 2018, the A-quality hybrid ‘Hymalaya’ and the milling hybrid ‘Hyena’ exhibited superior yield stability across the 52 environments of the German value for cultivation and use trials (www.bundessortenversuch.de). Remarkably, the A-quality line cultivar ‘Asory,’ which carries the T1AL.1RS rye translocation and which is part of our study, not only matched the performance of both hybrids in 2018 but also ranked third in yield stability out of 101 entries based on the ASR in the present study. This finding highlights the capacity of rye chromatin to contribute to yield stability through line breeding. Thus, our results both validate and extend previous conclusions by illustrating how modern breeding strategies leveraging alien introgression can narrow the hybrid–line gap in yield stability under variable environments.

### Integrating rye translocations into breeding for climate-resilient and productive winter wheat

As explained above, our results support an extended function for 1RS in breeding beyond its well-established source conferring disease resistance. The identification of translocation genotypes breaking traditional trade-offs, such as between yield and protein content or yield and stability, illustrates the potential within elite gene pools for complex trait improvement. However, rather than repositioning rye chromatin as a universal breeding solution, our findings emphasize that its contribution is context-dependent, shaped by the genomic background into which it is introgressed and by the environment in which it is expressed. The combination of genomic data with multi-environmental field-phenotyping underscores the importance of considering the interplay between introgressions, background, and environmental conditions. This integrative approach is essential for leveraging alien segments like 1RS as dynamic breeding tools rather than static trait carriers.

Going forward, 1RS translocations already available in the genetic background of elite germplasm represent one component of the genomic resources available for adaptation-oriented breeding. The lower stability variance observed for T1BL.1RS genotypes (Supplementary Fig. 3) indicates a more uniform response across environments; however, this uniformity coincided with significantly lower grain yield under drought. Thus, in this case, low variance reflects stable performance at a reduced level rather than improved drought resilience. In contrast, T1AL.1RS and especially the stacked T1AL.1RS/T1BL.1RS group combined higher yield under drought with only moderately increased variance, suggesting a more favorable productivity–stability balance. These findings underline that stability metrics must be interpreted together with the performance level. Low variance alone is not a sufficient breeding criterion. Breeding for resilience should therefore prioritize high and consistent performance under stress, not merely uniformity. Low WAASB or ASR values of genotypes with below-average grain yield represent static stability without agronomic relevance. In this case, the WAASBY superiority index allows identification of genotypes that are both productive and broadly adapted, which aligns directly with breeding goals.

Rank changes across water regimes similar to those observed in the present study have been reported for wheat panels when multiple drought indices were applied, confirming that G × E under drought is often expressed as crossover among indices (Semahegn et al. 2020). This underlines the need for selection in the target environment. The strong overlap between the top WAASBY performers and Cluster III genotypes identified by ASR cluster confirms the robustness of both approaches, while their occasional discrepancies underscore the nuanced trade-offs between performance-weighted and rank-based assessments of stability. The ASR-based clustering summarizes average ranks across multiple stability statistics and combines them with mean grain yield without imposing explicit weighting between stability and performance. In contrast, WAASBY integrates stability and yield using predefined weights, thereby aligning selection more directly with breeding priorities. In this framework, stability was consistently interpreted relative to yield level, recognizing that stable but low-yielding genotypes provide limited breeding value. The enrichment of 1RS translocation carriers, especially the placement of ‘Asory’ and ‘Debian’ among the top performers in the most stable and high-yielding cluster, demonstrates that rye chromatin, far from being a relic of stress-prone environments, offers broad agronomic value. These findings support a differentiated assessment of rye introgressions within climate adaptation strategies. Alien introgressions such as 1RS should be viewed not as universal stress-resistance factors but as genomic components whose value emerges through interaction with the polygenic wheat background.

The significant differences in stability behavior among genotype clusters (Supplementary Fig. 4) reveal that modern winter wheat still harbors a substantial diversity of responses to environmental variation. This finding indicates that breeding progress has not depleted adaptive variation but continues to generate distinct stability patterns among elite genotypes. Such diversity likely reflects the fundamental structure of wheat breeding itself. Considering that wheat is a self-pollinating species, breeders promote genetic renewal through recurrent crossing, followed by recombination and selection. This continuous reshuffling of alleles ensures that novel genotype combinations arise, providing the raw material for both broad and specific adaptability. Consequently, the diversity of responses observed among genotypes underscores the continuing long-term effectiveness of crossing-based breeding strategies in maintaining adaptability under changing environmental conditions. Through systematic crossing and selection, breeding accelerates allele reshuffling and facilitates adaptation to changing production environments.

To sustain such directed diversification, precise tools are required to track key introgressions and their variants. Here, we applied a multiplex SNP genotyping array enabling simultaneous identification of both T1BL.1RS and T1AL.1RS translocations in a broad panel of elite winter wheats. This array surpasses earlier marker systems by allowing efficient, high-throughput detection of 1RS variants within a genome-wide context. Notably, the platform uncovered rare genotypes with stacked 1RS translocations, offering new opportunities to harness underutilized rye alleles for breeding more resilient and productive wheat cultivars.

## Conclusion

This study demonstrates the value of combining molecular diversity analysis with multi-environment phenotyping in contrasting years to detect context-dependent effects of alien introgressions in modern winter wheat breeding germplasm. Our results show that the agronomic contribution of rye chromosome arm 1RS depends strongly on its interaction with the polygenic wheat background and environmental conditions. Rather than acting as a universally beneficial introgression, 1RS represents a genetic resource whose breeding value emerges from its integration into suitable genetic backgrounds.

The observed frequencies of T1AL.1RS (14.6%) and T1BL.1RS (21.7%) among modern German winter wheat germplasm indicate a moderate but targeted deployment of rye chromatin in contemporary breeding programs. Our results suggest that specific 1RS configurations can contribute to yield stability and stress resilience when strategically integrated into adapted germplasm. In the context of crop improvement, climate resilience is not limited to tolerance of a single stress factor such as drought. Rather, it refers to the ability of cultivars to maintain stable performance across highly variable and often contrasting environmental conditions. Modern wheat breeding programs therefore select genotypes that combine high yield potential with broad adaptation across diverse environments. The multi-environment evaluation presented in this study reflects this breeding framework and demonstrates that the agronomic value of alien introgressions such as rye chromosome arm 1RS should be interpreted in terms of performance stability across variable environments rather than under individual stress scenarios alone. At the same time, the 13.7% reduction in grain yield observed during the severe drought of 2022 highlights the continued sensitivity of elite winter wheat germplasm to extreme water limitation. Although modern wheat breeding programs rely on strong selection, they simultaneously maintain substantial genetic diversity through continuous recombination among elite germplasm and the targeted introgression of external genetic resources. Thus, breeding does not simply reduce diversity but actively reshapes and reassembles adaptive variation within elite populations. In this context, continued exploration of rye genetic diversity illustrates how systematic alien introgression can complement the recombination-driven diversity of modern wheat breeding to support the development of climate-resilient cultivars.

## Supplementary Information

Below is the link to the electronic supplementary material.

Supplementary file1 (DOCX 606 KB)

Supplementary file2 (XLSX 182 KB)

## Data Availability

The datasets generated and analyzed during the current study are included in this article and its Supplementary Material. Any additional information required to reproduce the analyses is available from the corresponding author on reasonable request.
