# Supplementary figures and images for: Introgression of rye chromosome arm 1RS enhances climate resilience in German winter wheat
Source: Theor Appl Genet. 2026 Jul 28;139(8):216. doi: 10.1007/s00122-026-05314-6 (PMC13415277; doi:10.1007/s00122-026-05314-6)

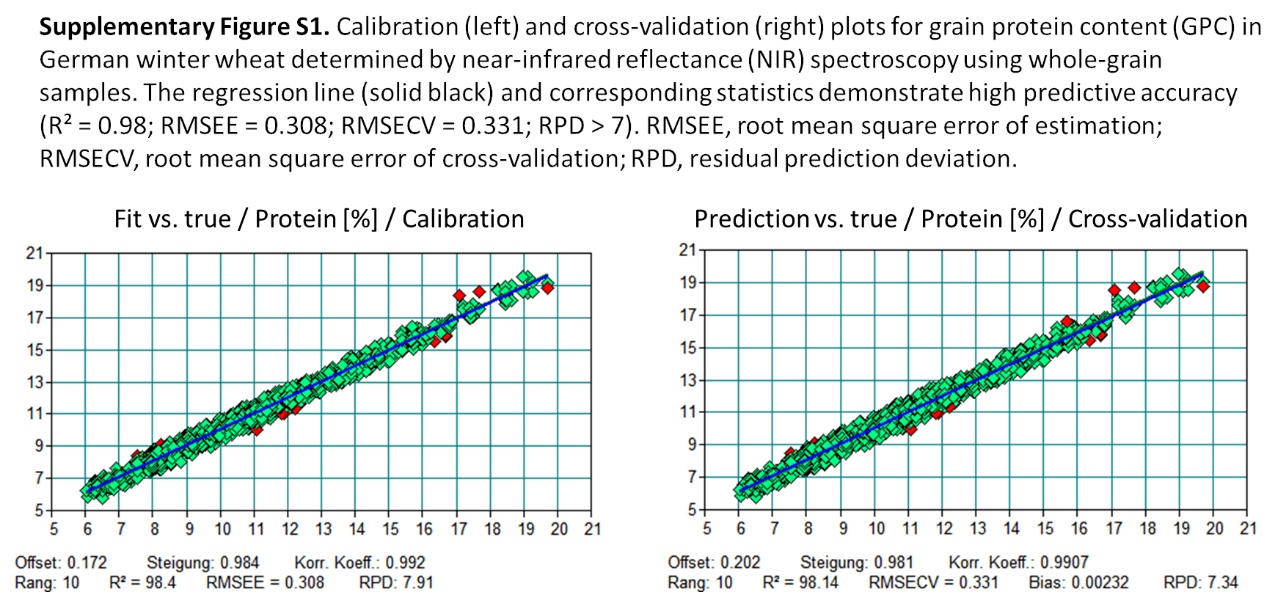


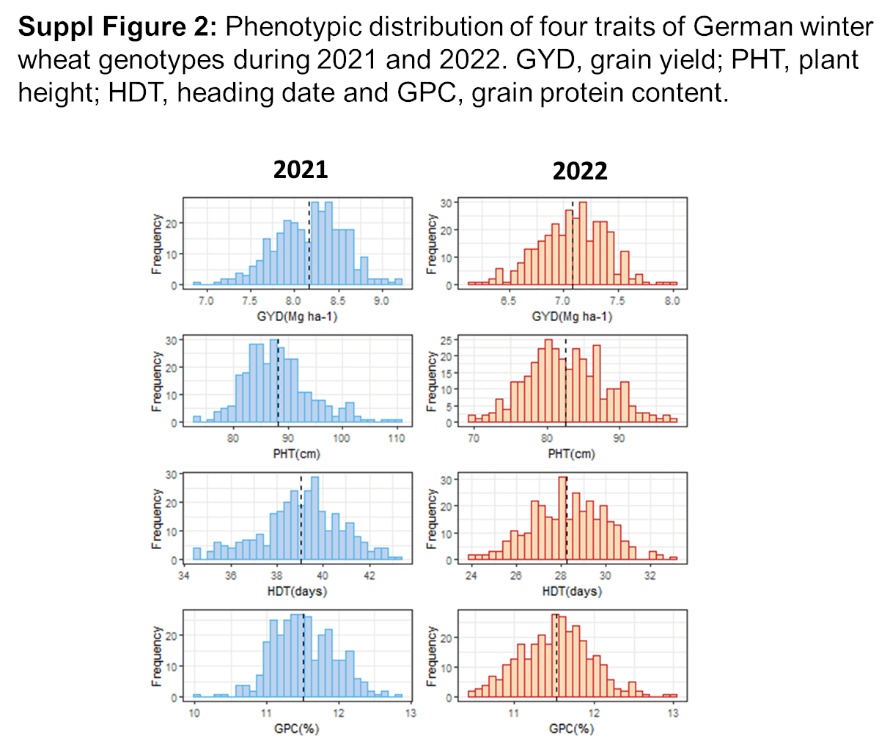


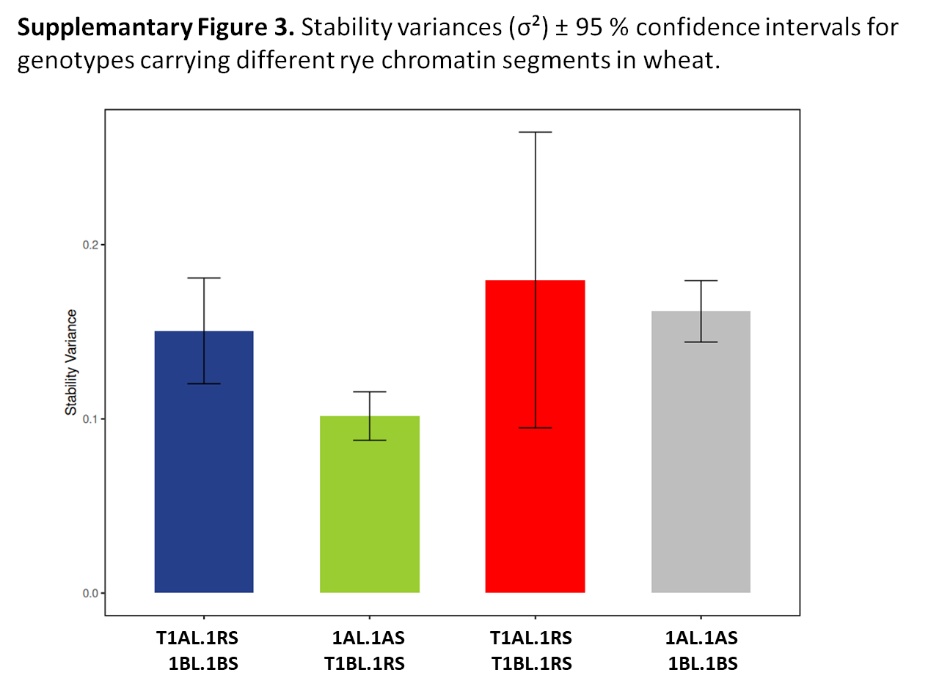

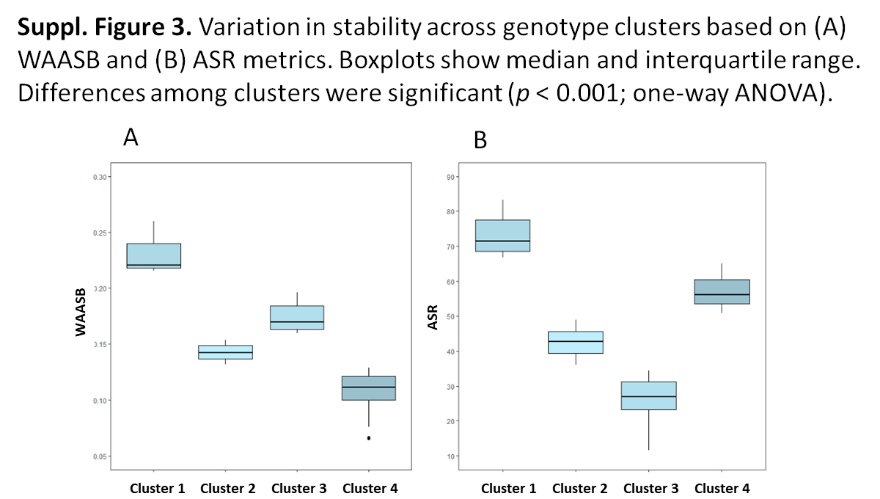

Supplement: Supplementary file 1 — Supplementary file1 (DOCX 606 KB) [file 122_2026_5314_MOESM1_ESM.docx]
